# Impact of COVID-19 lock-down period on orthopedic and trauma surgical activity in a northern Italian hospital

**DOI:** 10.3389/fmed.2024.1454863

**Published:** 2024-11-21

**Authors:** Marco Turati, Simone Gatti, Luca Rigamonti, Giovanni Zatti, Daniele Munegato, Marco Crippa, Erik Benedettini, Daniele Piscitelli, Marco Bigoni, Riccardo Turati

**Affiliations:** ^1^Orthopedic Department, Fondazione IRCCS San Gerardo dei Tintori, Monza, Italy; ^2^School of Medicine and Surgery, University of Milano-Bicocca, Monza, Italy; ^3^Transalpine Center of Pediatric Sports Medicine and Surgery, Monza, Italy; ^4^Orthopedic Department, Policlinico San Pietro, Ponte San Pietro, Italy; ^5^Department of Kinesiology, University of Connecticut, Storrs, CT, United States; ^6^Department of Applied Economics, Universitat Autonoma de Barcelona, Barcelona, Spain

**Keywords:** COVID-19, coronavirus, orthopedic, traumatology, lock-down

## Abstract

**Objectives:**

This study aims to describe the impact of COVID-19 on Orthopedic and Trauma surgical activity in a single level-I trauma center in Northern Italy during the lockdown period. We proposed comparing surgical procedures performed during the outbreak and in the same period the previous year.

**Methods:**

In this single-center retrospective epidemiological cohort study, the “lockdown cohort” of patients who were treated from March 1^st^ to May 24^th^, 2020, was compared to the “control cohort” who received treatment during the same period in 2019. The primary outcome was to evaluate the differences between the lockdown and control cohorts regarding surgical volumes. The secondary outcome was to evaluate any differences in the type of surgical procedures performed in the two cohorts in the elective and emergency setting.

**Results:**

Orthopedic surgical activity has suffered a global reduction of 72.4% during the lockdown period (from 36 ± 6.1 to 10.7 ± 8.4 per week; *p* < 0.01), with the ratio of emergency to elective operations increasing from 0.7:1 in 2019 to 3.3:1 in 2020. Elective surgery has in fact been almost completely suspended and was affected with a reduction of 88.9% (from 20.8 ± 5.2 to 4.3 ± 2.8 cases per week; *p* < 0.01), while emergency trauma surgery suffered a 49.7% reduction (from 15.1 ± 3.2 to 8.2 ± 6.1 cases per week; p < 0.01).

**Conclusion:**

The COVID-19 outbreak severely impacted Italy, particularly the Lombardy region, and affected the national health system. The 2020 COVID-19 lockdown has heavily conditioned our Orthopedic and Trauma department surgical activity.

## 1 Introduction

Following the outbreak of the epidemic in China, Italy—particularly the Lombardy region—emerged as the second epicenter of the novel coronavirus disease 2019 (COVID-19). The first Italian case was recorded in Codogno on February 21^st^, 2020, and from there, the virus spread rapidly throughout the region and the entire country (1). In response to this national emergency, the Italian government adopted two main strategies: health policies focused on strengthening hospital system capacity, and preventive measures like lockdowns and social distancing to reduce the risk of virus transmission (2).

On March 9th, the Italian government announced a national lockdown (hereinafter DPCM-1), and the Lombardy Regional Council issued a decree to guide the regional response to the outbreak, reorganizing the health system. To control the spread of the virus, approximately 90% of hospital departments were dedicated to treating COVID-19 patients. This reallocation of resources led to a reduction in the number of functioning operating rooms, allowing hospital staff (such as operating room nurses and anesthesiologists) and medical equipment, including ventilators, to be redeployed for COVID-19 care. Additionally, all licensed physicians, including those from the national army, were recruited to assist in the crisis (3).

The orthopedics department, which handles both elective and emergency services, also contributed to the pandemic response. Therefore, the measures implemented in the orthopedic field had to be adjusted to reorganize staff and equipment to address the challenges posed by COVID-19, while still maintaining emergency orthopedic services (4).

In this study, we aimed to investigate and analyze how the SARS-CoV-2 pandemic affected orthopedic services, particularly in terms of surgical activity in both elective and emergency settings. Our primary objective was to evaluate the differences in surgical volumes between the lockdown period and a control cohort. The secondary objective was to assess any changes in the types of surgical procedures performed during these periods (2019 vs. 2020) in both the elective and emergency settings.

## 2 Materials and methods

In this single-center retrospective epidemiological cohort study we reviewed the operating room activities of an Orthopedic and Trauma Department performed during the first Italian “wave” of COVID-19 pandemic in a single level-I trauma center in northern Italy (5).

Orthopedic operating room (OR) activities are divided into two categories: Trauma and Elective surgeries. Elective surgeries include scheduled orthopedic procedures such as arthroplasties, foot and ankle surgeries, hardware removal, nonunion treatments, pediatric elective procedures, and Sports Medicine interventions. Before surgery, all elective patients were evaluated in an outpatient setting, after which an orthopedic surgeon scheduled their procedure. Trauma surgeries, on the other hand, involve patients presenting through the emergency department who are indicated for surgery. Importantly, no changes to surgical guidelines occurred during this period.

All patients who underwent orthopedic surgery were included in the analysis, regardless of the department to which they were admitted, with no age restrictions applied. Only patients with incomplete data were excluded.

Data on patient characteristics (including age and gender), wait times from hospitalization to surgery, principal diagnoses, and the surgical procedures performed were retrieved from our computerized medical records. All data collection adhered to current Italian and European privacy laws and followed ethical guidelines for study conduct.

Both diagnoses and procedures are registered according to the International Classification of Diseases ICD 9th edition (ICD-9). Collected data are divided into two cohorts:

- The “lockdown cohort” included patients who underwent surgery at our hospital during the 12-week period from March 1st to May 24th, 2020, which coincided with the full lockdown in Lombardy.
- The “control cohort” consisted of patients who were surgically treated during the same 12-week period in the previous year, 2019. To account for variations in surgical activity on different days of the week, we used 1-week time spans (7 days of activity) as the unit of analysis. This approach allowed us to evaluate evolving trends during the pandemic and make comparisons with the previous year’s data.

**Figure 1:**
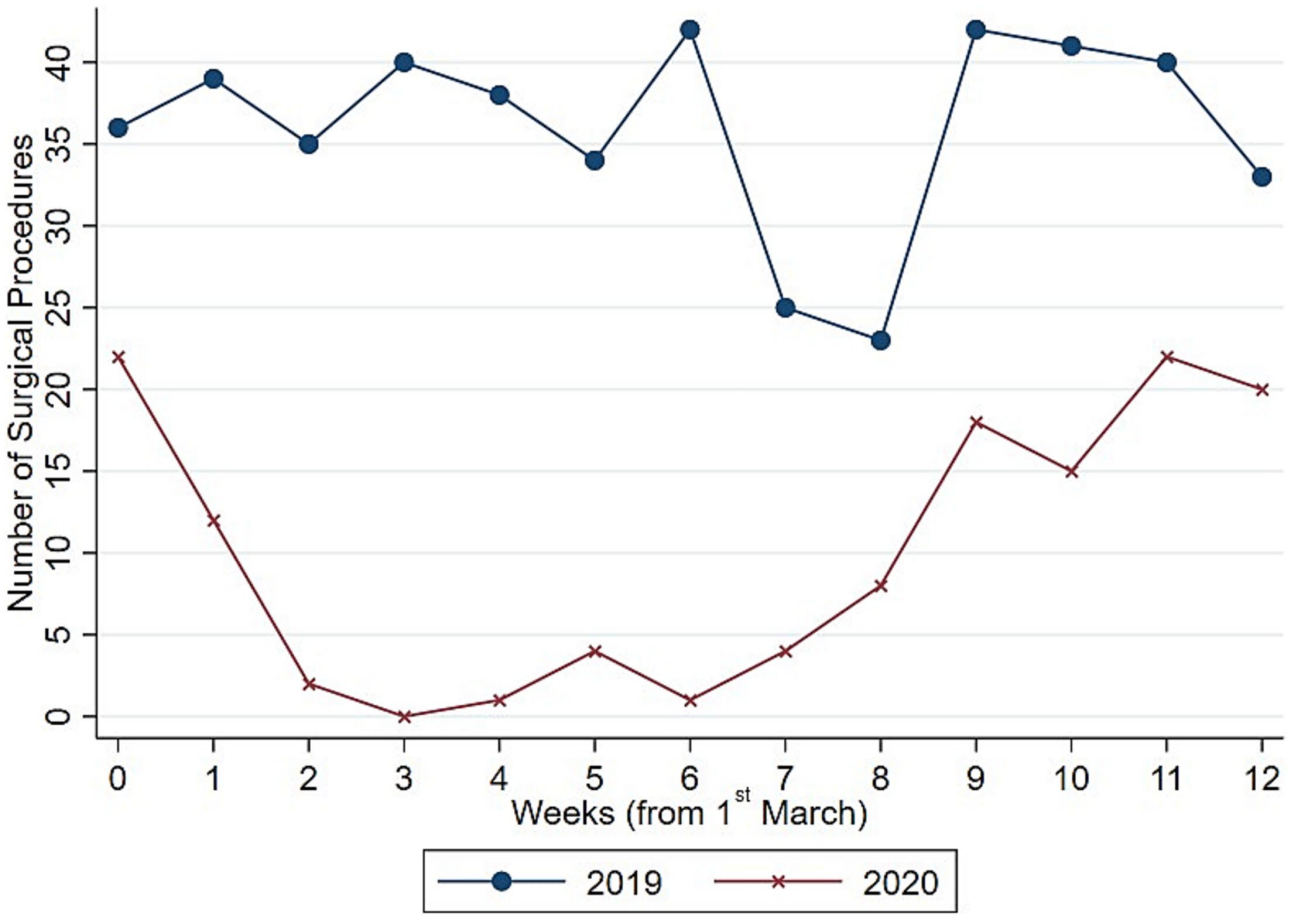
Evolution number of surgeries (total). Authors’ calculation overt Orthopedic and Trauma Department, Fondazione IRCCS San Gerardo dei Tintori Hospital, Monza (Italy). The Figure shows the weekly evolution in the Total Number of surgical procedures.

**Figure 2:**
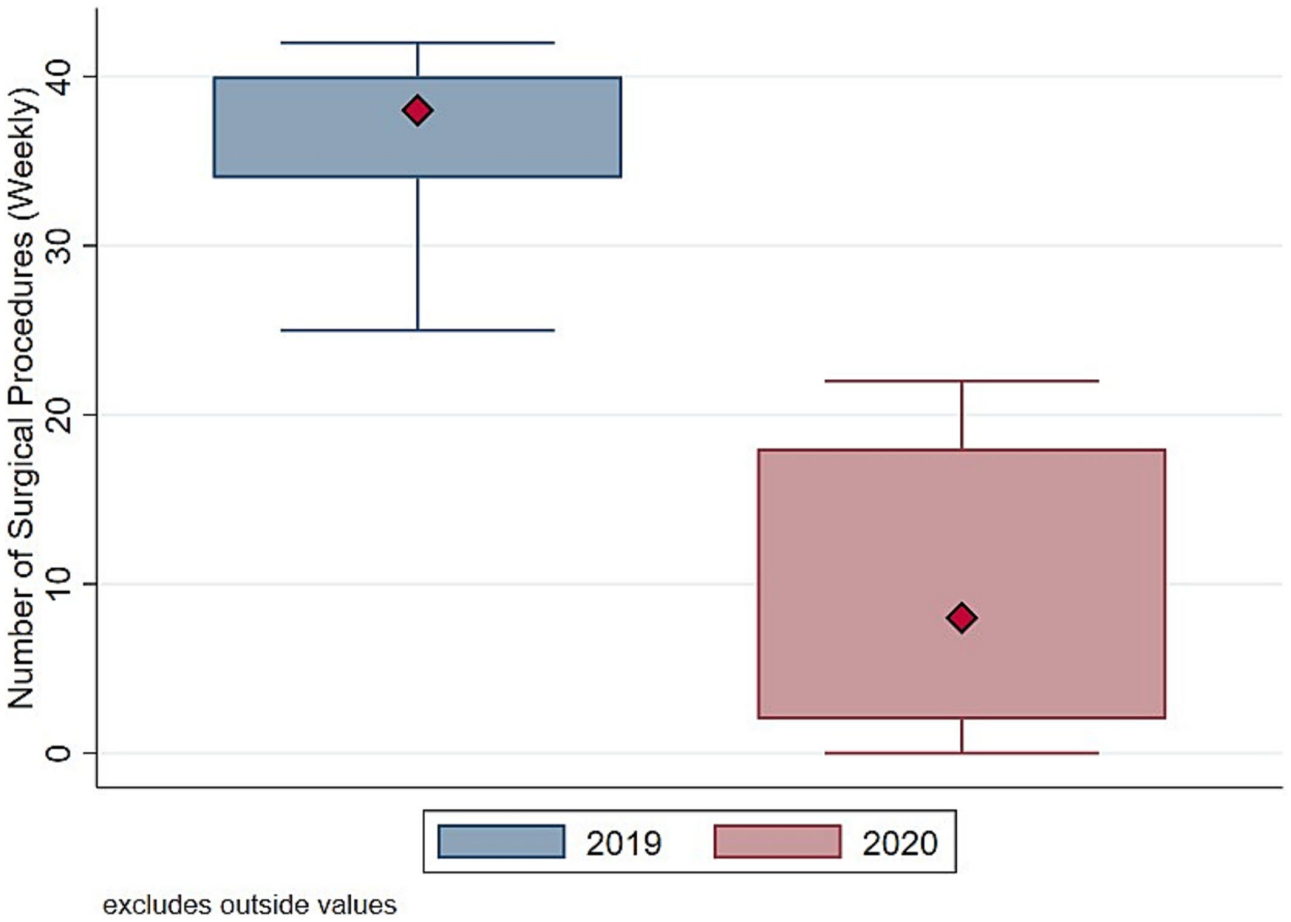
Distribution number of surgeries (total). Authors’ calculation overt Orthopedic and Trauma Department, Fondazione IRCCS San Gerardo dei Tintori Hospital, Monza (Italy). The Figure shows the boxplot of the distribution of weekly Total Number of surgical procedures.

Focusing on the total volume of surgical procedures, Figure 1 shows a significant decline in the weekly number of surgeries in 2020, with no procedures performed during week 3, which coincided with the first peak of COVID-19 cases at the end of March. Figure 2 highlights stark differences in the distribution of total surgeries over the entire period, with the median number of procedures in 2019 being nearly four times higher than in 2020. These findings highlight the relevant impact of the pandemic on surgical activities.

**Figure 3:**
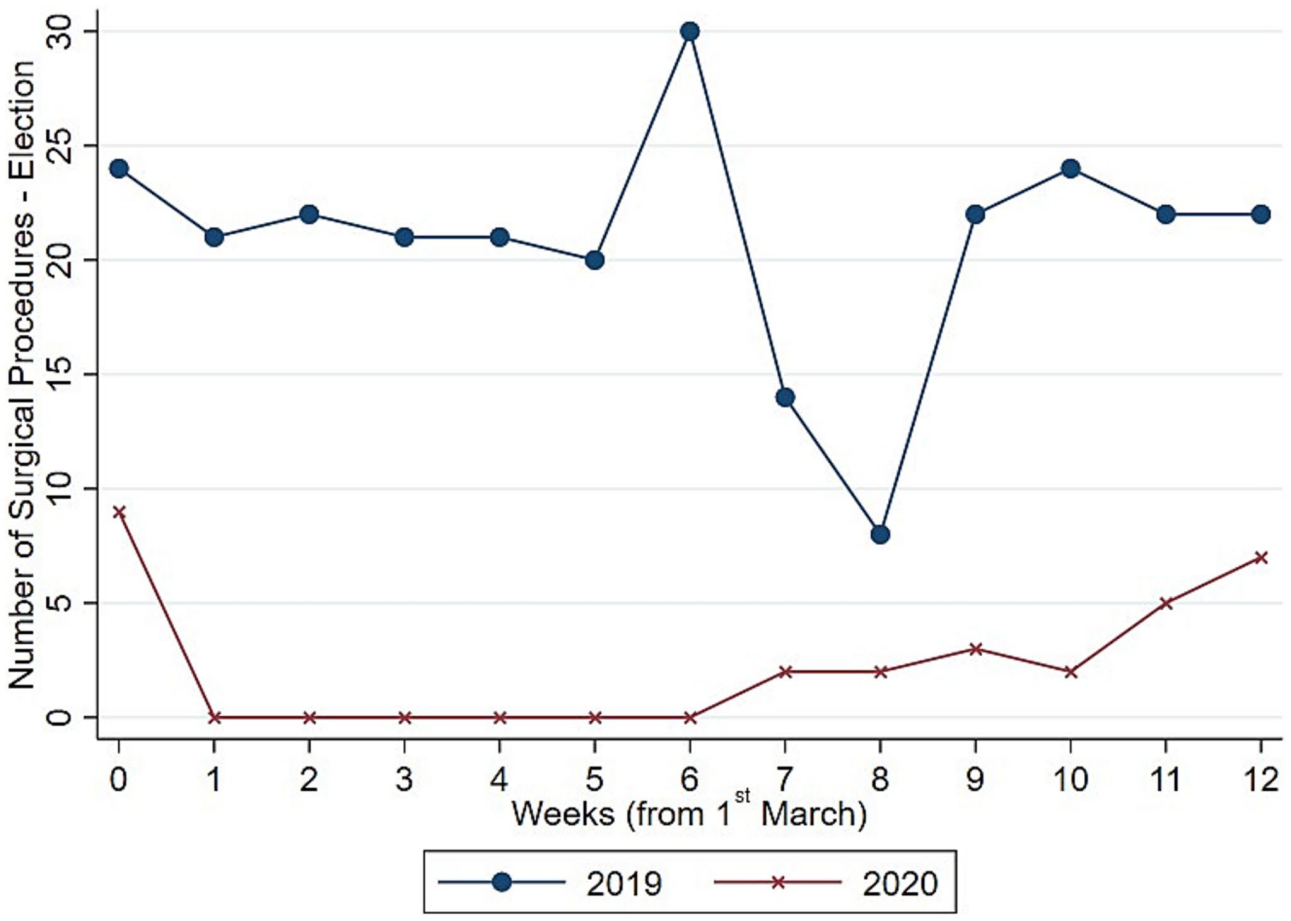
Evolution number of surgeries (elective). Authors’ calculation overt Orthopedic and Trauma Department, Fondazione IRCCS San Gerardo dei Tintori Hospital, Monza (Italy). The Figure shows the weekly evolution in the Number of Elective surgical procedures.

**Figure 4:**
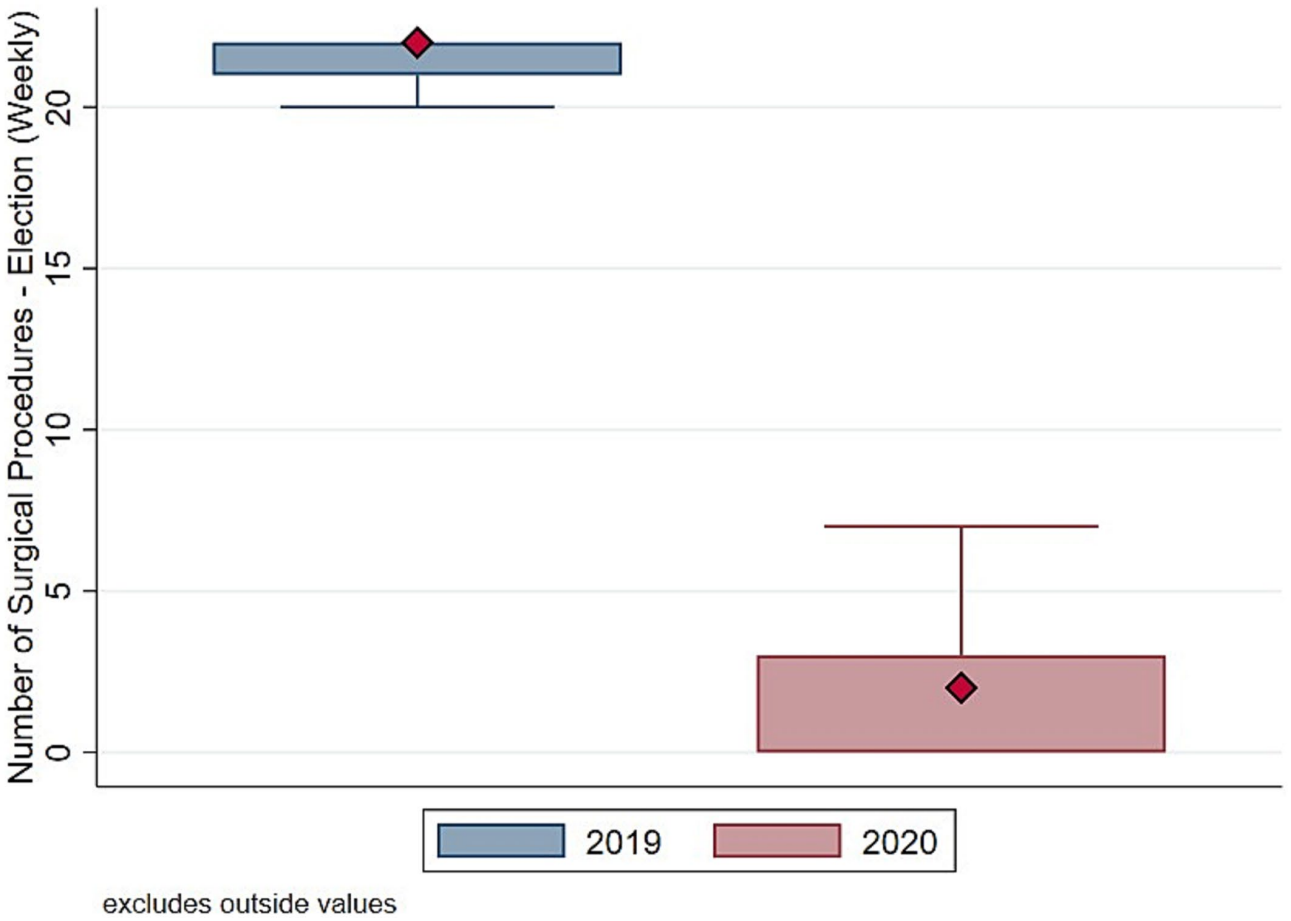
Distribution number of surgeries (elective). Authors’ calculation overt Orthopedic and Trauma Department, Fondazione IRCCS San Gerardo dei Tintori Hospital, Monza (Italy). The Figure shows the boxplot of the distribution of weekly Number of Elective surgical procedures.

**Figure 5:**
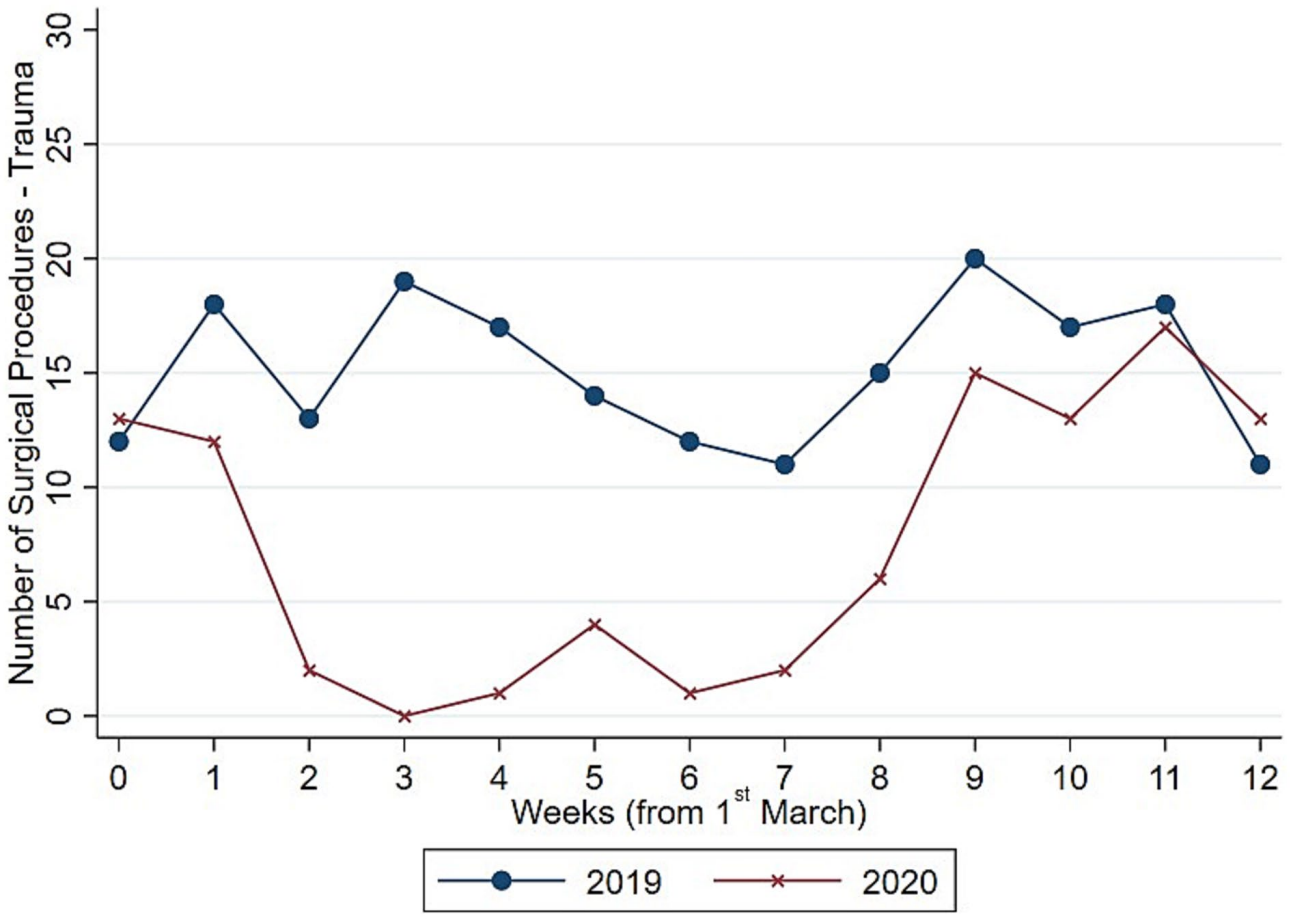
Evolution number of surgeries (trauma). Authors’ calculation overt Orthopedic and Trauma Department, Fondazione IRCCS San Gerardo dei Tintori Hospital, Monza (Italy). The Figure shows the weekly evolution in the Number of Trauma surgical procedures.

**Figure 6:**
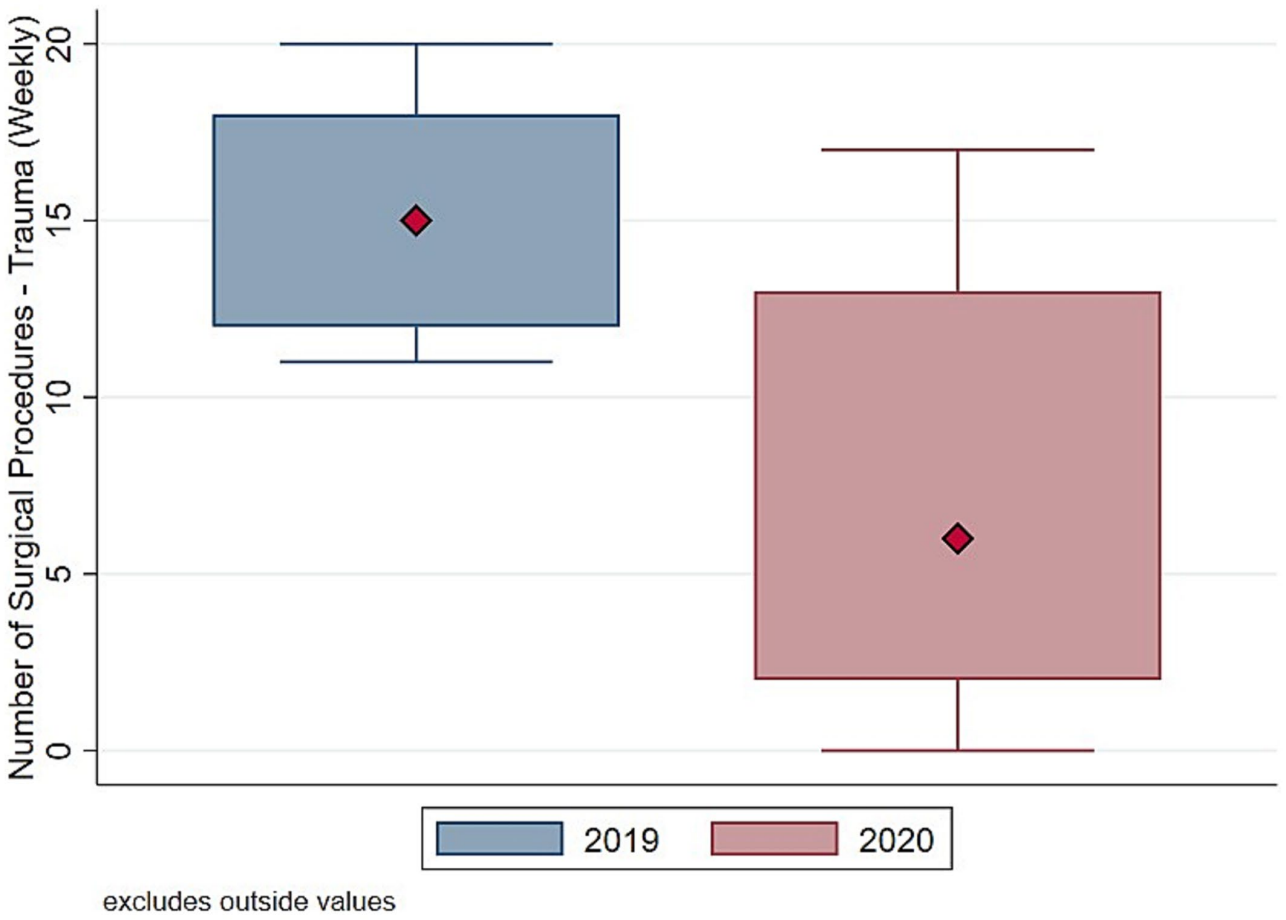
Distribution number of surgeries (trauma). Authors’ calculation overt Orthopedic and Trauma Department, Fondazione IRCCS San Gerardo dei Tintori Hospital, Monza (Italy). The Figure shows the boxplot of the distribution of weekly Number of Trauma surgical procedures.

To understand whether such drop in surgical procedure influenced homogeneously the activity of the Orthopedic and Trauma center, we decompose the evolution and the distribution of surgical procedures between elective (Figures 3, 4) and trauma (Figures 5, 6) procedures. The results show stark differences in elective surgeries between the lockdown and control cohorts, whereas the differences in trauma procedures between 2019 and 2020 are less pronounced.

**Figure 7:**
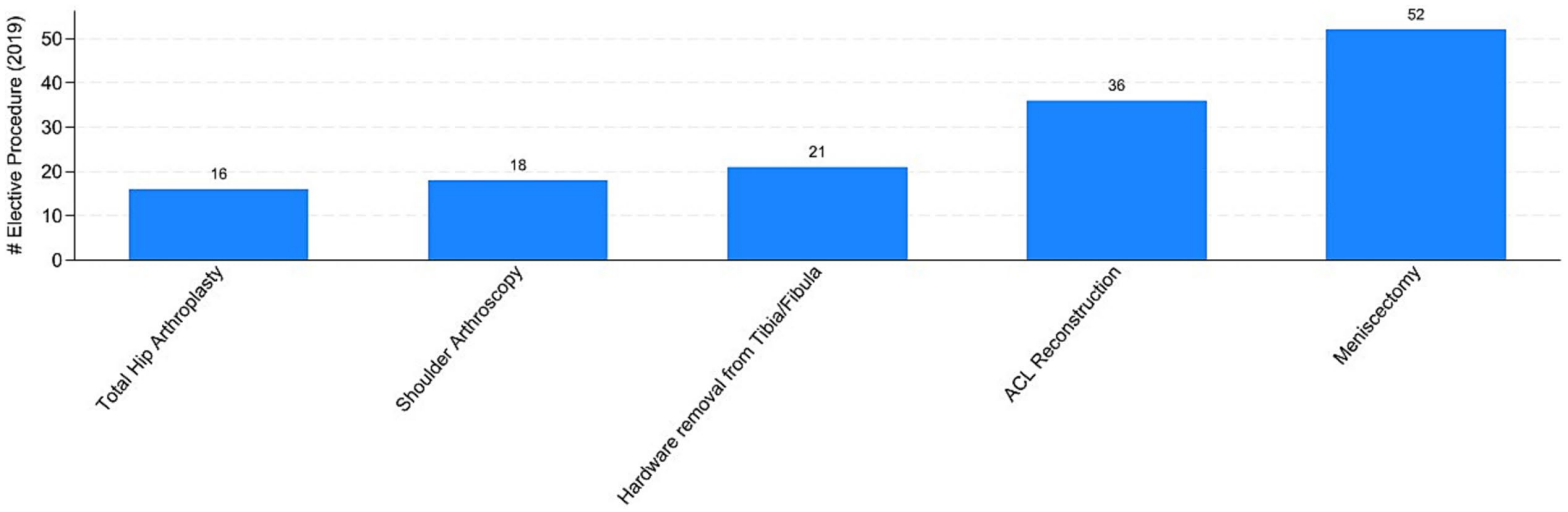
Top-5 elective procedures in 2019. Authors’ calculation overt Orthopedic and Trauma Department, Fondazione IRCCS San Gerardo dei Tintori Hospital, Monza (Italy). The Figure reports the top 5 elective surgical procedure in 2019. ACL stands for “Anterior Cruciate Ligament.”

**Figure 8:**
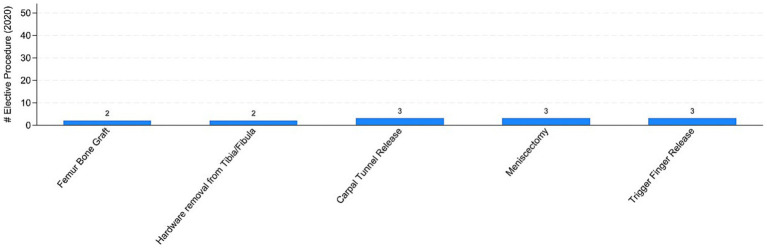
Top-5 elective procedures in 2020. Authors’ calculation overt Orthopedic and Trauma Department, Fondazione IRCCS San Gerardo dei Tintori Hospital, Monza (Italy). The Figure reports the top 5 elective surgical procedure in 2020.

**Figure 9:**
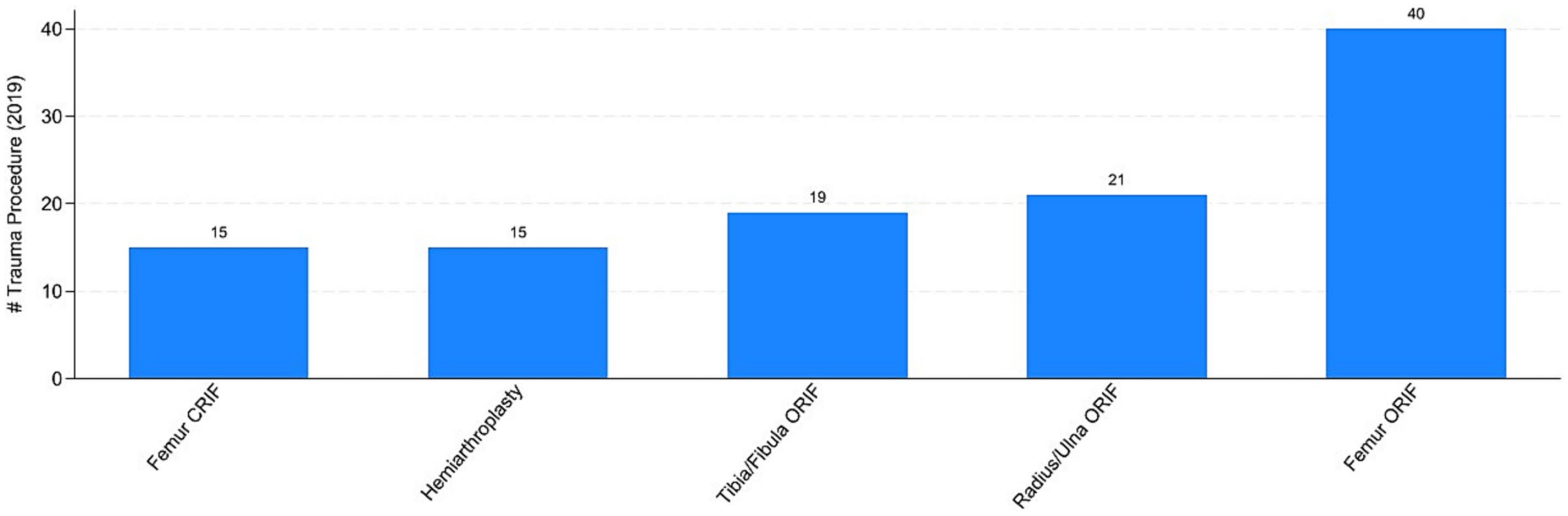
Top-5 trauma procedures in 2019. Authors’ calculation overt Orthopedic and Trauma Department, Fondazione IRCCS San Gerardo dei Tintori Hospital, Monza (Italy). The Figure reports the top 5 trauma surgical procedure in 2019. ORIF stands for “Open Reduction and Internal Fixation” and CRIF stands for “Closed Reduction and Internal Fixation.”

**Figure 10:**
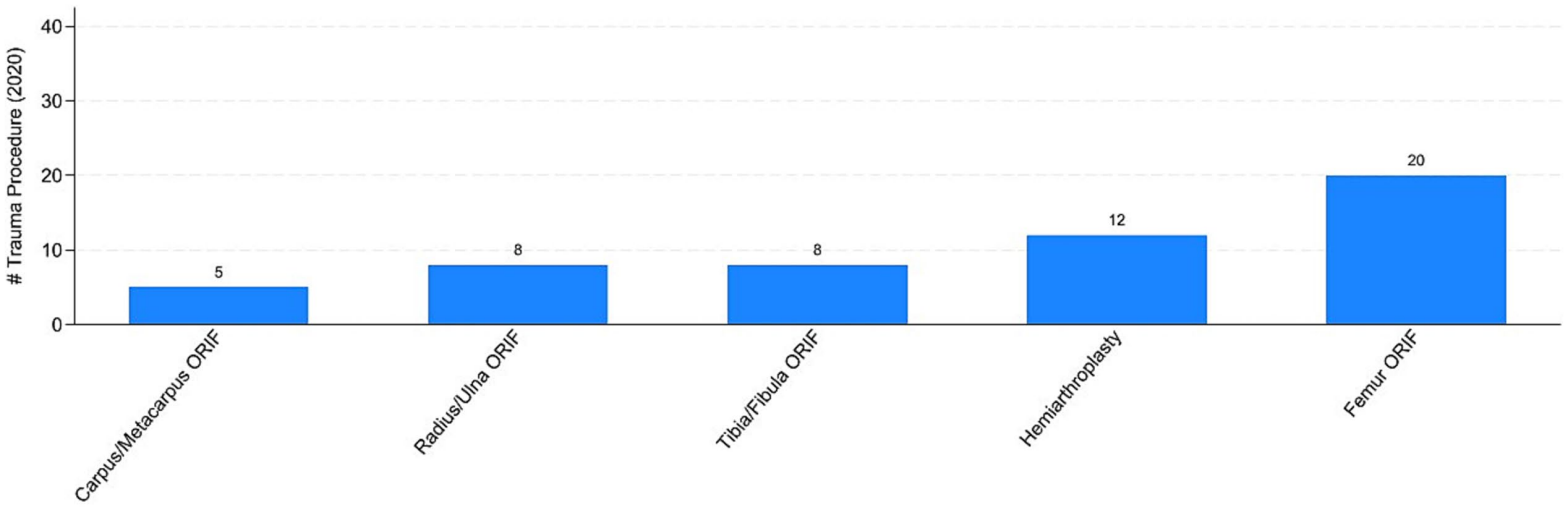
Top-5 trauma procedures in 2020. Authors’ calculation overt Orthopedic and Trauma Department, Fondazione IRCCS San Gerardo dei Tintori Hospital, Monza (Italy). The Figure reports the top 5 trauma surgical procedure in 2020. ORIF stands for “Open Reduction and Internal Fixation.”

The COVID pandemic influenced not only the volume of surgical procedures, but also the type of procedures. To illustrate this, we analyzed the five most performed (i.e., “Top 5”) procedures in 2019 and 2020, distinguishing between elective and trauma surgeries. In the elective field, there was a drastic reduction in both the volume and variety of procedures. Comparing Figures 7, 8 the number of joint replacement surgeries and arthroscopic procedures dropped by 90%. For instance, the meniscectomy, the most common elective surgery in 2019, was performed only three times in 2020. In contrast, as shown in Figures 9, 10 the types of trauma procedures remained relatively consistent between 2019 and 2020. The most frequently performed surgery in both cohorts was open reduction and internal fixation (ORIF) for femoral fractures (26.4% of all procedures in 2019 and 19.8% in 2020). In the lockdown cohort, hemiarthroplasty ranked second (12.1%), followed by ORIF for tibia and fibula fractures (8.1%), ORIF for ulna and radius fractures (8.1%), and ORIF for carpal-metacarpal fractures (5.0%). In 2019, after femur fracture surgeries, the next most common trauma procedures were ORIF for ulna and radius fractures (10.6%), ORIF for tibia and fibula fractures (9.6%), and hemiarthroplasty (7.6%).

Overall, these stylized facts provide three key pieces of evidence: (i) the total number of surgical procedures was significantly lower in 2020 compared to 2019, (ii) the reduction was not uniform across elective and trauma procedures, and (iii) the types of elective procedures changed between 2019 and 2020, while this was less evident for trauma procedures. In the following section, we present our empirical strategy to validate these findings, controlling for confounding factors and the distribution of the outcome variables.

### 2.1 Statistical analysis

Our statistical analysis, conducted using STATA-16 for Windows, aims to determine whether the COVID-19 lockdown influenced (i) the volume of orthopedic surgical procedures and (ii) the types of procedures performed. After aggregating our individual data by week, we first compared the average number of surgical procedures between 2019 and 2020. To assess whether the differences in the number of procedures between these 2 years are statistically significant, we conducted a t-test and reported the results. Additionally, we examined whether average patient characteristics (age and gender) and the time from hospitalization to surgery differed between the lockdown cohort and the control cohort, ensuring that variations in the patient population did not account for differences in surgical activity.

To simultaneously control for confounding factors, we estimated the following linear model:


(1) $$Y_{w}=\alpha +\beta Covid^{2020}+\Gamma X_{w}+\epsilon_{w}$$

where our outcome variable $Y_{w}$ is the number of surgical procedure (total, elective, trauma) or the number of surgical procedures by type of procedure (e.g., Orhopaediatrics, Arthroplasty, etc.) for each week w ∈ {0, 1, …, 12}. The variable of interest is $Covid^{2020}$ , which is a dummy variable that takes value of one in the year 2020. The vector $X_{w}$ includes a set of controls that can influence the volume and type of surgical procedure. The vector includes the average patient age, gender composition, and the average number of days between hospitalization to surgery. Finally, our model accounts for potential heteroskedasticity of the error term $\epsilon_{w}$ .

Estimating Equation 1 with a standard ordinary least square (OLS) estimator provides the estimates of the partial correlation between the lockdown period and surgical procedures $\left(\hat{\beta }\;\right)$ For instance, if the dependent variable is the total number of surgical procedures, a negative $\hat{\beta }\;$ suggests that in the lockdown period the weekly number of surgical procedures is smaller than the control period, after controlling for patient characteristics. Due to the substantial number of zeros in the dependent variable, OLS estimator can fit less precisely our data (6). For this reason, we follow (7) and we also estimate our baseline using the Poisson pseudo maximum likelihood estimator (hereafter PPML). Such an estimator is well suited to address large mass of zeros in the dependent variable and heteroskedasticity patterns in the error terms.

## 3 Results

### 3.1 Surgical volumes

From the early days of the outbreak, Fondazione IRCCS San Gerardo dei Tintori Hospital began reorganizing all departmental activities to address the emergency. Following the implementation of the lockdown on March 8^th^, 2020, more restrictive measures were adopted, including the suspension of all non-urgent surgical and outpatient services. Table 1 presents the total number of orthopedic and trauma procedures performed in 2019 and 2020. During the 61-day lockdown period (from March 1st to April 30th, 2020), a total of 129 cases were treated, compared to 468 cases during the same period in 2019. This represents a decrease of 339 cases, equating to a drop of approximately 72.4% (from an average of 36 ± 6.1 cases per week to 10.7 ± 8.4 cases per week; *p* < 0.01).

**Table 1:** Surgical procedures.

	(1)	(2)	(3)	(4)	(5)	(6)
Year	Total No.	Trauma	Trauma (%)	Day(s) surg.	Age	Women (%)
2019	468	197	42.1	2.6	51.7	45.5
2020	129	99	76.7	2.6	55.8	48.1

Authors’ calculations on data from Orthopedic and Trauma Department, Fondazione IRCCS San Gerardo dei Tintori Hospital, Monza (Italy). Columns (1)–(2) shows the total number of surgical procedures and trauma-specific, respectively. Column (3) presents the percentage of trauma-specific over the total. Column (4) shows the average number of days between the hospitalization and the surgery. Column (5) presents the average age of the patients, while column (6) shows the percentage of female patients.

The reduction in orthopedic surgical volume is not proportionate across operative categories. Table 2 shows the differences across total, elective and trauma surgeries. Elective surgery has been almost completely suspended and it is the most affected with an 88.9% reduction (from 20.8 ± 5.2 to 4.3 ± 2.8 per week; *p* < 0.01). Emergency Trauma surgery suffers “only” a 49.7% reduction (from 15.1 ± 3.2 to 8.2 ± 6.1 per week; *p* < 0.01). Most of orthopedic surgical activities in the lockdown period consisted of emergency Trauma procedures (76.7%), with a Trauma-to-Elective (T:E) ratio of 3.3:1. Before the pandemic our control cohort registered a T:E ratio of 0.7:1, with Trauma surgery representing only 42.1% of total orthopedic OR activity. Notably, there were no significant differences in the demographic characteristics (sex and age) of the two patient cohorts, as shown in Table 2, indicating that variations in patient characteristics do not fully account for these differences. However, the number of days between hospitalization and surgery decreased in the emergency setting, dropping from an average of 5.2 days in 2019 to 2.1 days in 2020 (*p* < 0.05).

**Table 2:** Surgical procedures—weekly statistics.

Type of Surgical Proc.	Total			Elective			Trauma		
	(1)	(2)	(3)	(4)	(5)	(6)	(7)	(8)	(9)
	2019	2020	Difference	2019	2020	Difference	2019	2020	Difference
Number	36 (6.096)	10.75 (8.400)	-25.25^***^ (-8.65)	20.85 (5.178)	4.286 (2.812)	-16.56^***^ (-7.80)	15.15 (3.184)	8.250 (6.107)	-6.904^***^ (-3.59)
Patients’ Age	51.59 (6.072)	58.10 (12.64)	6.504 (1.66)	45.91 (10.08)	47.14 (14.54)	1.231 (0.22)	59.39 (5.463)	60.27 (15.29)	0.872 (0.19)
Women (share)	0.454 (0.0677)	0.575 (0.253)	0.121 (1.66)	0.438 (0.154)	0.482 (0.339)	0.0440 (0.40)	0.495 (0.126)	0.593 (0.282)	0.0986 (1.14)
Day(s) Surg.	2.668 (1.474)	3.529 (5.544)	0.861 (0.54)	0.763 (0.900)	6.304 (14.66)	5.541 (1.39)	5.189 (3.040)	2.142 (1.477)	-3.047^**^ (-3.14)
Observations	468	129	597	271	30	301	197	99	296

Authors’ calculation on data from Orthopedic and Trauma Department, Fondazione IRCCS San Gerardo dei Tintori Hospital, Monza (Italy). The table shows the weekly average number of surgical procedures, patients’ age, share of women and days between hospitalization and surgical operation over the years 2019 and 2020, and the differences between the 2 years over the total surgical procedures [col. (1)–(3)], over the elective surgical procedures [col. (4)–(6)] and over the trauma surgical procedures [col. (7)–(9)]. Standard errors are reported in parenthesis in columns (1), (2), (4), (5), (7), and (8) while p-values are reported in parenthesis in columns (3), (6), and (9). Level of significance: ^*^p < 0.1, ^**^p < 0.05, ^***^p < 0.01.

Finally, Table 3 presents the estimates from our benchmark Equation 1. The outcome variable includes the weekly total number of procedures [columns (1)–(4)], elective procedures [columns (2)–(5)], and trauma procedures [columns (3)–(6)]. Focusing on the OLS estimates [columns (1) to (3)], the results indicate that the lockdown significantly reduced the total number of orthopedic cases: on average, there were 25 fewer cases per week compared to the same period in 2019, including 18 elective cases and nearly 7 trauma cases. These coefficients are precisely estimated at the 1% significance level. The results are confirmed using a non-linear estimator (PPML) in columns (4) to (6). By interpreting the coefficients as semi-elasticities and controlling for confounding factors, we find that the pandemic decreased the weekly total number of orthopedic procedures by 71.1%. As expected, the results vary considerably between elective and trauma procedures: elective procedures were reduced by 88.4%, while trauma procedures experienced a milder reduction of 47.6% due to the pandemic.

**Table 3:** Surgical procedures—regression results.

Estimation	OLS			PPML		
Surgical procedures	Total	Elective	Trauma	Total	Elective	Trauma
	(1)	(2)	(3)	(4)	(5)	(6)
COVID^2020^	−25.121^***^ (3.171)	−18.301^***^ (1.952)	−6.821^***^ (1.929)	−1.243^***^ (0.225)	−2.158^***^ (0.361)	−0.646^***^ (0.209)
Age	0.254 (0.157)	0.046 (0.085)	0.209^**^ (0.100)	0.017 (0.012)	0.006 (0.014)	0.024^*^ (0.013)
Women	−18.194 (11.264)	−4.215 (5.964)	−13.980^*^ (7.496)	−1.218 (0.885)	−0.834 (1.252)	−1.545^*^ (0.892)
Day(s) Surg.	−0.125 (0.217)	−0.012 (0.109)	−0.113 (0.171)	−0.015 (0.026)	−0.005 (0.032)	−0.019 (0.030)
Observations	26	26	26	26	26	26
*R* ^2^	0.80	0.84	0.51	0.76	0.82	0.46

Authors’ calculation on data from Orthopaedic and Trauma Department, Fondazione IRCCS San Gerardo dei Tintori Hospital, Monza (Italy). Robust standard errors are reported in parenthesis. Level of significance: ^*^p < 0.1, ^**^p < 0.05, ^***^p < 0.01. The results report the coefficients from: OLS estimator [col. (1)–(3)], and PPML estimator [col. (4)–(6)]. The R2 associated to the PPML estimations is the standard Pseudo *R*^2^.

### 3.2 Surgical procedures

Regarding the secondary outcome of the study, we assessed whether the pandemic influenced not only the total number of procedures but also affected the types of procedures conducted differently. Figures 7, 8 indicate that the most commonly performed elective procedures changed significantly between 2019 and 2020, while trauma procedures showed less variation. In this section, we test this observation by presenting estimates based on our empirical model outlined in Equation 1, after aggregating elective and trauma procedures into broad categories.

Concerning elective procedures, Table 4 presents the results on the number of procedures, aggregated into six broad categories: arthroplasty (col. 1), foot and ankle (col. 2), hardware removal (col. 3), non-unions and infections (col. 4), orthopediatrics (col. 5), and sports medicine (col. 6). Linear estimates are shown in the top panel, while the bottom panel presents non-linear estimates.

**Table 4:** Elective surgical procedures—regression results.

Type of procedure	Arthroplasty	Foot and ankle	Hardware removal	Non-unions infections	Orthopediatrics	Sports medicine
	(1)	(2)	(3)	(4)	(5)	(6)
Panel A: OLS results						
COVID^2020^	−1.414^***^ (0.495)	−0.536 (0.421)	−0.640 (0.426)	−0.585 (0.366)	−2.310^**^ (0.915)	−4.421^*^ (2.158)
Age	0.027^**^ (0.010)	0.045^**^ (0.019)	0.047^*^ (0.025)	0.027^**^ (0.009)	0.100^*^ (0.054)	0.094^*^ (0.048)
Women	−0.411 (0.785)	−1.016 (1.246)	−1.263 (1.487)	1.325 (0.860)	−0.125 (2.445)	1.473 (2.007)
Day(s) Surg.	−0.001 (0.006)	0.017 (0.023)	−0.481 (0.506)	−0.025 (0.049)	3.502 (2.807)	−1.724 (2.340)
Observations	26	26	26	26	26	26
*R* ^2^	0.81	0.64	0.57	0.59	0.60	0.89
Panel B: PPML results						
COVID^2020^	−1.166^***^ (0.273)	−1.336 (1.057)	−2.824^**^ (1.175)	−0.940^**^ (0.435)	−1.924^***^ (0.313)	−2.713^***^ (0.572)
Age	0.046^***^ (0.007)	0.037^***^ (0.014)	0.027^**^ (0.012)	0.037^***^ (0.007)	0.111^***^ (0.034)	0.022^***^ (0.008)
Women	−0.093 (0.292)	−0.605 (0.746)	−0.509 (0.583)	1.589^***^ (0.568)	0.138 (0.762)	1.755^**^ (0.728)
Day(s) Surg.	0.009^***^ (0.003)	0.003 (0.010)	0.642 (0.403)	−0.014 (0.031)	0.624 (0.784)	1.951^***^ (0.750)
Observations	26	26	26	26	26	26
Pseudo *R*^2^	0.79	0.47	0.46	0.53	0.56	0.90

Authors’ calculation on data from Orthopaedic and Trauma Department, Fondazione IRCCS San Gerardo dei Tintori Hospital, Monza (Italy). Robust standard errors are reported in parenthesis. Level of significance: ^*^p < 0.1, ^**^p < 0.05, ^***^p < 0.01. Panel A reports the OLS estimates, while Panel B reports PPML estimates.

Focusing on the OLS results, we observe some degree of heterogeneity across procedures. During the pandemic, there were approximately 4.4 fewer procedures per week related to sports medicine (*p* < 0.1), 2.3 fewer for orthopediatrics (*p* < 0.05), and 1.4 fewer associated with arthroplasty (*p* < 0.01). Results for foot and ankle interventions, hardware removal, and non-unions/infections were not statistically different from zero. However, the lack of precision in some estimates may stem from the considerable number of zeros in the dependent variable, which can create estimation issues for a linear model. Indeed, the coefficients related to the pandemic period estimated from the non-linear model presented in panel B are all statistically significant, except for those associated with foot and ankle procedures.

Interpreting the estimates as semi-elasticities, elective procedures dropped by 68.9% for arthroplasty, 94.1% for hardware removal, 60.9% for non-unions/infections, 85.4% for orthopediatrics, and 93.4% for sports medicine during the lockdown. Overall, these results indicate that the lockdown influenced the various types of elective procedures differently.

Table 5 presents the linear and non-linear estimates associated with trauma surgical procedures, aggregated into the following seven groups: elbow, wrist, and hand fractures (col. 1); hip and femur fractures (col. 2); knee, ankle, and foot fractures (col. 3); orthogeriatric procedures (col. 4); pediatric procedures (col. 5); polytrauma (col. 6); and shoulder and upper arm fractures (col. 7). Interestingly, the estimated coefficients are not statistically significant or are only marginally significant (*p* < 0.1) in a few cases. These results appear to confirm the evidence presented in Figures 9, 10. While there was a decrease in the total number of trauma cases, the types of procedures remained relatively consistent between 2019 and 2020.

**Table 5:** Trauma Surgical procedures—regression results.

Type of procedure	Elbow wrist and hand fractures	Hip and femur fractures	Knee ankle and foot fractures	Orthogeriatric	Pediatric	Polytrauma	Shoulder and upper arm fractures
	(1)	(2)	(3)	(4)	(5)	(6)	(7)
Panel A: OLS results							
COVID^2020^	1.423 (0.937)	−0.146 (0.465)	−0.331 (0.509)	−1.222 (0.783)	−0.134 (0.236)	−0.457 (0.291)	0.008 (0.353)
Age	0.045^***^ (0.010)	0.044^***^ (0.009)	0.046^***^ (0.012)	0.023^*^ (0.012)	0.152^***^ (0.026)	0.027^***^ (0.008)	0.053^**^ (0.020)
Women	0.980 (0.811)	−1.439^**^ (0.612)	−1.140 (0.819)	1.148 (1.012)	−0.186 (0.349)	−0.291 (0.656)	−1.679 (1.300)
Day(s) Surg.	0.102 (0.071)	−0.028 (0.054)	0.048 (0.050)	0.081 (0.159)	−0.500^***^ (0.165)	0.046^**^ (0.018)	−0.092 (0.127)
Observations	26	26	26	26	26	26	26
R^2^	0.58	0.54	0.54	0.41	0.72	0.67	0.50
Panel B: PPML results							
COVID^2020^	0.761^*^ (0.430)	−0.194 (0.316)	−0.071 (0.346)	−0.370^*^ (0.217)	0.007 (0.391)	−0.778^*^ (0.418)	0.314 (0.460)
Age	0.039^***^ (0.009)	0.035^***^ (0.008)	0.039^***^ (0.009)	0.038^***^ (0.011)	0.196^***^ (0.038)	0.036^***^ (0.008)	0.075^***^ (0.015)
Women	0.178 (0.320)	−0.721^**^ (0.288)	−1.159^**^ (0.464)	0.371 (0.313)	−0.058 (0.429)	−0.365 (0.444)	−1.817^**^ (0.748)
Day(s) Surg.	0.075^*^ (0.044)	−0.018 (0.027)	0.051^*^ (0.027)	0.052 (0.037)	−0.538^**^ (0.223)	0.024^***^ (0.008)	−0.051 (0.065)
Observations	26	26	26	26	26	26	26
Pseudo *R*^2^	0.47	0.42	0.39	0.37	0.58	0.59	0.48

Authors’ calculation on data from Orthopaedic and Trauma Department, Fondazione IRCCS San Gerardo dei Tintori Hospital, Monza (Italy). Robust standard errors are reported in parenthesis. Level of significance: ^*^p < 0.1, ^**^p < 0.05, ^***^p < 0.01. Panel A reports the OLS estimates, while Panel B reports PPML estimates.

## 4 Discussion

Our study demonstrates the significant impact of COVID-19 on elective and emergency orthopedic surgery. Given the unpredictable nature of the SARS-CoV-2 epidemic, particularly during the first wave, the lockdown policy implemented by the Italian government resulted in an overall reduction of approximately 70% in surgical volumes, dropping from 468 cases in 2019 to 129 cases in 2020. The reduction was more pronounced for elective surgeries compared to trauma emergency services (88.9% vs. 45.7%). During the initial weeks of the pandemic, international guidelines for orthopedic surgeons recommended minimizing in-hospital stays for all orthopedic patients and postponing any surgical procedures that could be deferred (8). Only non-deferrable conditions in the emergency setting, such as fractures and bone infections requiring urgent management, were admitted and treated accordingly. Notably, our data indicate a reduction in the number of days between hospitalization and surgery, decreasing from an average of 5.2 days in 2019 to 2.1 days in 2020 (*p* < 0.05). However, these results can be partially explained by the redirection of residual surgical capacity toward managing these specific conditions and the reduction in in-hospital length of stay.

During the initial weeks of the pandemic, international guidelines for orthopedic surgeons recommended reducing the in-hospital length of stay for all orthopedic patients and postponing any surgical procedures that could be deferred (9, 10). Additionally, the decline in trauma-related admissions during the COVID-19 outbreak may be attributed to a reduction in public activities, fewer traffic-related accidents, and a decrease in sports injuries due to restrictive social distancing measures, such as the closure of gyms and sports clubs during the national lockdown (11, 12). Furthermore, the fear of contracting the virus may have deterred patients from presenting to emergency departments with minor traumas, contributing to the overall reduction in hospital admissions (4).

An epidemiological study conducted in Italy reported a significant drop in the incidence of proximal femoral fractures nationwide between 2019 and 2020. As illustrated in Figures 9, 10 our study corroborates this decline, with femoral fractures remaining the most frequently performed procedures (13).

The delay in treating non-urgent orthopedic surgeries represented a burden that the Italian health system is partially still facing (14). These restrictions also affected the diagnostic process, resulting in delays in both the diagnosis and subsequent treatment of several orthopedic conditions (15). Additionally, patients with confirmed diagnoses were unable to access treatment promptly. Consequently, the implementation of specific treatment protocols, which consider timeliness in the decision-making process, could not be realized (16, 17). The experiences of various medical centers have demonstrated that telemedicine could serve as a valuable tool to facilitate the diagnostic process. Further investigation is needed to equip patients and physicians with new, effective instruments for improving care (18), and to better explore the long-lasting consequences of such health shock.

Notably, Italian government regulations during this period also affected other emergency surgical services (19). Furthermore, an intriguing retrospective observational study revealed that the COVID-19 pandemic significantly impacted the volume of surgical procedures performed in a rural hospital. However, despite a shift towards non-operative management for conditions such as appendicitis and acute cholecystitis, there was no long-term increase in severe cases requiring surgery (20). The scientific community’s research agenda should focus on investigating the extent to which changes in patient care plans during the COVID-19 pandemic affected health outcomes. The experiences of worldwide healthcare service reductions may provide valuable insights into minimizing unnecessary care (21).

The first wave of the pandemic exposed the world’s unpreparedness to handle such an emergency. Since then, several improvements have been made. Flexibility and adaptability, along with scientific and technological advancements, have emerged as crucial factors in addressing this situation. Additionally, postoperative management has undergone various changes; the use of telemedicine has become a vital tool, enabling the monitoring of patients during rehabilitation without requiring them to visit a specialist in the hospital (22). Moreover, the COVID-19 pandemic highlighted the importance of primary care in ensuring continuity of care following hospital discharge.

In conclusion, the COVID-19 outbreak severely impacted the entire Italian national health system. Significant efforts were needed in terms of restrictions and the mobilization of healthcare workers, which hindered the ability to maintain standard care for various diseases. Our Orthopedic and Trauma department faced substantial challenges, leading to a significant reduction in all services, including emergency department consultations, outpatient consultations, and surgeries. Our experience may offer valuable insights for the future; however, addressing a future pandemic will require multidisciplinary coordination.

## Data Availability

The raw data supporting the conclusions of this article will be made available by the authors, without undue reservation.
